# Assess the Performance and Cost-Effectiveness of LACE and HOSPITAL Re-Admission Prediction Models as a Risk Management Tool for Home Care Patients: An Evaluation Study of a Medical Center Affiliated Home Care Unit in Taiwan

**DOI:** 10.3390/ijerph17030927

**Published:** 2020-02-02

**Authors:** Mei-Chin Su, Yi-Jen Wang, Tzeng-Ji Chen, Shiao-Hui Chiu, Hsiao-Ting Chang, Mei-Shu Huang, Li-Hui Hu, Chu-Chuan Li, Su-Ju Yang, Jau-Ching Wu, Yu-Chun Chen

**Affiliations:** 1 Department of Nursing, Taipei Veterans General Hospital, Taipei 11217, Taiwan; mcsu@vghtpe.gov.tw (M.-C.S.); shchiu2@vghtpe.gov.tw (S.-H.C.); huangms@vghtpe.gov.tw (M.-S.H.); ccli2@vghtpe.gov.tw (C.-C.L.); lhhu@vghtpe.gov.tw (L.-H.H.); sryang@vghtpe.gov.tw (S.-J.Y.); 2 Institute of Hospital and Health Care Administration, National Yang-Ming University, Taipei 11221, Taiwan; tjchen@vghtpe.gov.tw; 3 Department of Family Medicine, Taipei Veterans General Hospital, Taipei 11217, Taiwanhtchang2@vghtpe.gov.tw (H.-T.C.); 4 Department of Primary Care and Public Health, Imperial College London, London W6 8RP, UK; 5 School of Medicine, National Yang-Ming University, Taipei 11221, Taiwan; jauching@gmail.com; 6 Department of Pediatric Neurosurgery, Neurological Institute, Taipei Veterans General Hospital, Taipei 11217, Taiwan

**Keywords:** home care patient, readmission, prediction model, simulation, risk management

## Abstract

The LACE index and HOSPITAL score models are the two most commonly used prediction models identifying patients at high risk of readmission with limited information for home care patients. This study compares the effectiveness of these two models in predicting 30-day readmission following acute hospitalization of such patients in Taiwan. A cohort of 57 home care patients were enrolled and followed-up for one year. We compared calibration, discrimination (area under the receiver operating curve, AUC), and net reclassification improvement (NRI) to identify patients at risk of 30-day readmission for both models. Moreover, the cost-effectiveness of the models was evaluated using microsimulation analysis. A total of 22 readmissions occurred after 87 acute hospitalizations during the study period (readmission rate = 25.2%). While the LACE score had poor discrimination (AUC = 0.598, 95% confidence interval (CI) = 0.488–0.702), the HOSPITAL score achieved helpful discrimination (AUC = 0.691, 95% CI = 0.582–0.785). Moreover, the HOSPITAL score had improved the risk prediction in 38.3% of the patients, compared with the LACE index (NRI = 0.383, 95% CI = 0.068–0.697, *p* = 0.017). Both prediction models effectively reduced readmission rates compared to an attending physician’s model (readmission rate reduction: LACE, 39.2%; HOSPITAL, 43.4%; physician, 10.1%; *p* < 0.001). The HOSPITAL score provides a better prediction of readmission and has potential as a risk management tool for home care patients.

## 1. Introduction

Prediction of readmission has received a lot of research attention recently for its important role in quality improvement and evolving payment reforms [1,2,3]. Previous studies suggest that readmissions occurred frequently in 15–25% of patients and are associated with excess harm and numerous burdens to the health system [3,4]. The LACE index and the HOSPITAL score are two of most commonly used prediction models that proactively stratify and identify patients at a high risk of readmission [5,6,7,8,9]. The simplicity of the LACE index and HOSPITAL score distinguishes them from other models, and they have been widely adopted in current clinical information systems as a result [9]. However, quite a few studies found that either the LACE index or HOSPITAL score, originally developed for medical and surgical patients, demonstrates inconsistent model performance when used in different settings [10,11,12,13].

Prediction of readmission is critically important to reduce readmissions for home health care patients. The readmission rate is high, around 30–100% among such patients, and greatly impacts the whole health system [3,14]. Those home care patients usually have a certain degree of disability and are quite vulnerable after leaving the hospital; such a significant jump between the hospital and home requires preventive interventions with more insight into patient health at home. An ideal readmission prediction model helps to stratify patients with a high risk of readmission, targets the delivery of resource-intensive interventions to the patients with the greatest need, and improves outcomes [15].

There is limited information on using the LACE index and HOSPITAL score models as risk assessment tools against readmission for home care patients. As there is no single prediction model that works well in all scenarios, most readmission prediction models require validation and evaluation using real world data in different settings [7,9]. The goal of this study is to evaluate how well the LACE index and HOSPITAL score models predict whether home care patients will be readmitted after their hospitalization. Moreover, a microsimulation model is performed to analyze and compare the cost and effectiveness of readmission prediction models using real-world data.

## 2. Materials and Methods

### 2.1. Setting, Data Source, and Ethical Concerns

This retrospective cohort study was conducted in a medical center affiliated with a home care unit providing a large service of more than 5000 home visits for 400 disabled patients per year in Taipei, Taiwan. Since several factors increase the likelihood of readmission, the current setting of this study helped to minimize influences resulting from system-level factors and to focus on patient-level factors. The complexity of transition between hospital and home care service is largely simplified by a team effort (patient transferal for hospitalization by home-visit physician, intra-hospital discharge planning, and post-discharge instruction by home care nurses), which minimizes the bias resulting from external factors. Moreover, the high availability of Taiwan’s health system further reduces any bias introduced regarding the concern of accessibility barriers for disabled patients.

Demographic information and relevant variables were extracted from the hospital’s electronic medical system. This study’s protocol was approved by the Institutional Review Board of Taipei Veterans General Hospital (IRB#2018-08-016AC). The Review Board waived the requirement of written informed consent from each patient who was involved in our study because the data we analyzed were de-identified secondary data, which were published for research purposes. No identifiable private information or human biospecimens were involved.

### 2.2. Study Cohort, Enrolled Hospitalizations, and 30-Day Readmissions

Patients who were registered and used home care services in this home care unit before 31 December 2016 constituted the study population. In Taiwan, patients who are wheelchair- or bed-bound for more than half of the day or who require considerable assistance and frequent medical care are eligible for home care services covered by Taiwan’s National Health Insurance.

**Figure 1:**
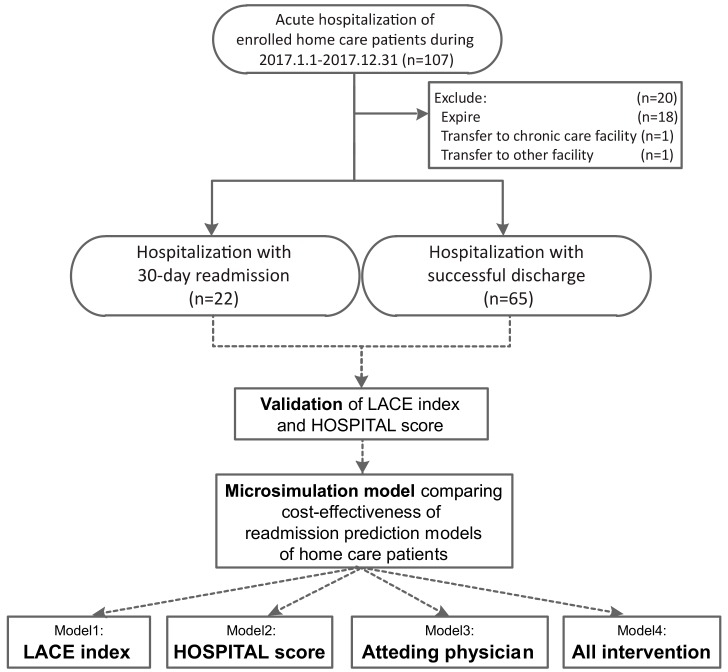
Flowchart of data processing for outcomes of acute hospitalization of a disabled cohort at a medical center affiliated home care unit in Taiwan, 2017 (*n* = 107).

We enrolled all acute hospitalizations between 1 January 2017 and 31 December 2017 as the study population. We excluded acute hospitalization which met the following conditions: end of the study, transfer to a chronic care facility (e.g., nursing home, respiratory care ward), transfer to other home care facilities, or death. Multiple eligible hospitalizations made by a single patient was counted accordingly (Figure 1).

The outcomes of discharge of enrolled acute hospitalizations were determined by 30-day readmissions. Hospitalizations were designated as “hospitalization with 30-day readmissions” if patients were readmitted within 30 days after their discharge, whereas hospitalizations were regarded as “hospitalization with successful discharge” if they were free from readmission. Readmission rates were calculated as the number of “hospitalization with 30-day readmissions” divided by the number of hospitalizations.

### 2.3. The LACE Index and HOSPITAL Score Models for Prediction of Readmission

To compare the performance of the LACE index and HOSPITAL score models, both scores were calculated for each hospitalization (Figure 1).

The LACE indexes were calculated by points assigned for the patient’s length of stay, the acuity of the patient’s admission, the degree of comorbid illness or illnesses (as measured by the Charlson comorbidity index), and the number of times patients had been to the emergency department in the last six months [16].

The HOSPITAL scores were calculated by points assigned for blood hemoglobin level at discharge, blood sodium level at discharge, having a procedure during the hospital stay, index admission type, number of hospital admissions in the previous year, and length of stay of more than 5 days. We did not include the factor “discharge from an oncology service” since such groups rarely used oncology-related services because of limited benefits. For patients with multiple blood test results during hospitalization, the last one closest to the discharge date was used [17].

**Figure 2:**
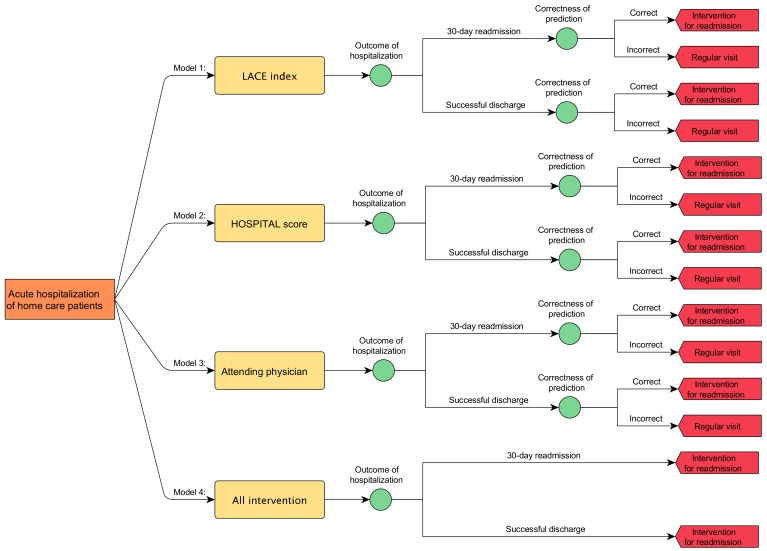
Microsimulation model to compare cost-effectiveness of four readmission prediction models when applied to acute hospitalizations of a disabled cohort of a medical center affiliated home care unit in Taiwan, 2017. (*n* = 87).

Since there was no single best measurement for the performance of a prediction model, we compared the LACE and HOSPITAL models with the following measurements: discrimination, calibration, sensitivity, specificity, and net reclassification improvement (NRI) [18]. A microsimulation model, shown below in Figure 2, simulated and further evaluated the cost-effectiveness of each model before being applied to the real world. Discrimination is the ability of a predictive model to separate hospitalizations into high risk of 30-day readmission or successful discharge. Measured as an area under the receiver-operating curve (AUC), it is suggested that an AUC less than 0.60 reflects poor discrimination; 0.60 to 0.75, possibly helpful discrimination; and more than 0.75, clearly useful [19]. Calibration, however, as a measure reflects the extent to which a model correctly estimates the absolute risk (i.e., the difference between predicted and observed risk at different levels).

NRI is a new and popular method for assessing how much better a new model is at predicting risk than another model; it is called net reclassification. NRI is usually expressed as the percentage of a new model reclassifying patients into risk categories compared with a previous model [20].

### 2.4. Microsimulation Model

Microsimulation is a technique used to model complex real-life events by simulating the impact of policy change on the individual units (micro units) that make up the system where the events occur [21,22]. We constructed a microsimulation model to evaluate the cost-effectiveness of the LACE index and HOSPITAL score when applied to hospitalizations of the current study cohort. To estimate the effect of prediction in the real world, we incorporated a third model, attending physician, as a 30-day readmission prediction made by home-care attending physicians as a ground truth. According to a previous study, the sensitivity and specificity of an attending physician were 0.23 (95% CI = 0.13–0.37) and 0.84 (95% CI = 0.75–0.90), respectively [23]. The fourth model, all intervention (no prediction, null model), was introduced by assuming preventive efforts were performed for all acute hospitalizations without any prediction.

We assumed the home care unit should make a preventive intervention against readmission for every acute hospitalization when predicted with a high risk for 30-day admission after discharge to home. The cost of interventions thus equals the number of hospitalizations predicted as high risk for 30-day admission multiplied by the unit cost of the intervention. A 30-day readmission reduction rate is calculated by the difference between observed and expected readmission rates. Naturally, a better model has a lower cost of interventions and a higher readmission reduction rate. However, the cost of physicians (i.e., extra payment for physicians doing prediction work) and prediction model deployment (i.e., maintenance cost for machines and technicians) are not counted in the simulation model.

### 2.5. Error Analysis and Statistical Analysis

Error analysis was performed to examine the measurement error of the data. The first author (M.-C.S.) rechecked the data and verified with an electronic health record. Statistical analyses were performed using IBM SPSS Statistics, version 24, (IBM Corp., Armonk, NY, USA), MedCalc, version 19 (MedCalc Software, Ostend, Belgium), and R, version 3.6.1 (R Foundation for Statistical Computing, Vienna, Austria). Microsimulation was performed using TreeAge Pro 2019, R2 (TreeAge Software, Williamstown, MA, USA). Demographic information, admission history, and parameters for LACE and HOSPITAL scores were obtained from the electronic medical record. Receiver operator characteristic (ROC) curves were constructed for the LACE model and the HOSPITAL model and Youden’s index was used to determine the cut-off point of both models. We compared calibration with the Hosmer–Lemeshow goodness-of-fit test and discrimination with an area under the receiver operating curve (AUC). The reclassification with net reclassification improvement (NRI) was used to identify patients at risk of 30-day readmission for both models. A two-tailed level of 0.05 was considered statistically significant.

### 2.6. Experimentation of Microsimulation Model

A microsimulation analysis was performed to assess the cost-effectiveness of readmission prediction models. Performance measures (sensitivities and specificities) and readmission rates calculated from the real-world data were used as variables in the microsimulation model (Appendix A, Table A1 and Table A2). We further assumed preventive efforts intervened for every high-risk hospitalization (i.e., acute hospitalizations predicted as a high likelihood of readmission) with a success rate of 50%. The current model only included the direct costs of preventive intervention against readmission for acute hospitalization for those predicted as at high risk for readmission, thus the cost of a physician (e.g., extra payment) and maintenance of a computer system were not counted. Cost of the all intervention model (i.e., no prediction model) was used as a reference. Cost-effectiveness pair with scatter plot was used to evaluate these four readmission prediction models.

## 3. Results

### 3.1. Study Population and 30-Day Readmission Rates

A total of 87 acute hospitalizations of a home care patient cohort of a medical center affiliated home care unit in 2017 was included in the current study. Generally, the study population was quite old, at an average age of 87.4 years. The readmission rate was 25.3%, which means more than a quarter of patients were readmitted within 30 days after their discharge. There was no age and gender difference between hospitalizations with 30-day readmission and with successful discharge. While HOSPITAL scores of hospitalizations with 30-day readmission were significantly higher than hospitalizations with successful discharge, there was no significant difference for the LACE index between these two outcomes of hospitalization (Table 1). The error analysis showed that the issue of measurement error is minimal in the current study.

### 3.2. Discrimination and Calibration of the LACE and HOSPITAL Models

**Figure 3:**
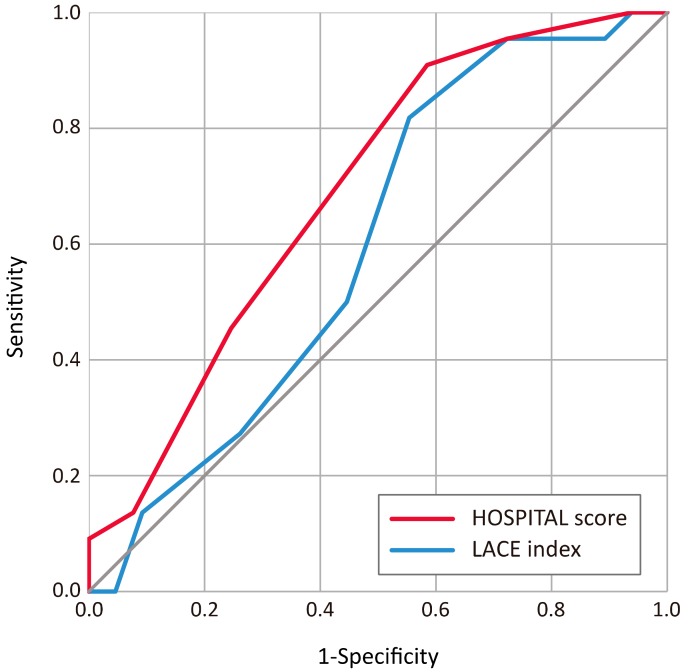
Receiver operating characteristic (ROC) curves of the LACE index and HOSPITAL score prediction models for 30-day readmission after acute hospitalization of a home care patient cohort of a medical center affiliated home care unit in Taiwan, 2017 (*n* = 87).

HOSPITAL scores achieved helpful discrimination (AUC = 0.691, 95% CI = 0.582–0.785), while LACE scores had poor discrimination (AUC = 0.598, 95% CI = 0.488–0.702) (Figure 3, Table 2). The findings suggest that HOSPITAL scores have a better ability to identify high risk readmission hospitalization. Both LACE or HOSPITAL scores show agreement between observed readmission and predictions (Hosmer–Lemeshow goodness-of-fit test, both *p* > 0.05). There is a high agreement between LACE index and HOSPITAL score (number of agreements vs. disagreements: 69 vs. 18; McNemar test, *p* = 1.000) which suggests that the results of both models are similar.

The LACE index and HOSPITAL score models show similar performance in terms of accuracy, sensitivity, and specificity (Table 3). However, the HOSPITAL score improved the risk prediction in 38.3% of the patients, compared with the LACE index (NRI = 0.383, 95% CI = 0.068-0.697, *p* = 0.017), which clearly suggests the HOSPITAL score model is 38.3% more likely to identify hospitalization with high risk of 30-day readmission than the LACE index.

### 3.3. Cost of Intervention and Readmission Rate Reduction of the LACE Index and HOSPITAL Score Models

Both the LACE index and HOSPITAL score models are useful as risk assessment tools against readmission among home care patients, although the HOSPITAL score performs slightly better than the LACE index model. These two models effectively reduce the readmission rate compared to attending physicians, with the cost of more preventive efforts. There was a clear 30-day readmission rate reduction of 40% for both the LACE index and HOSPITAL score models. Such findings suggest that the LACE index and HOSPITAL score models significantly reduce readmission rates much more than attending physicians (readmission rate reduction of the LACE index model vs. attending physician model, 39.2% vs. 10.1%; the HOSPITAL score model vs. attending physician model, 43.4% vs. 10.1%; both *p* < 0.001). Moreover, the HOSPITAL score model further reduced the readmission rate by 4% compared to the LACE index (readmission rate reduction of the LACE index vs. the HOSPITAL score, 39.2% vs. 43.4%, *p* < 0.05 when sample size >1100) (Table 3).

However, the simulated results clearly show that the all intervention model (no prediction) is the costliest model, followed by the HOSPITAL score and LACE index models, respectively. In contrast, the attending physician model has the least cost. The cost of preventive efforts of the LACE index and HOSPITAL score models was at least 3.5 times larger than that of the attending physician model (LACE index vs. attending physician, 66.8% vs. 18.6%, 3.58-fold; HOSPITAL score vs. attending physician, 72.0% vs. 18.6%, 3.87-fold) (Table 3).

**Figure 4:**
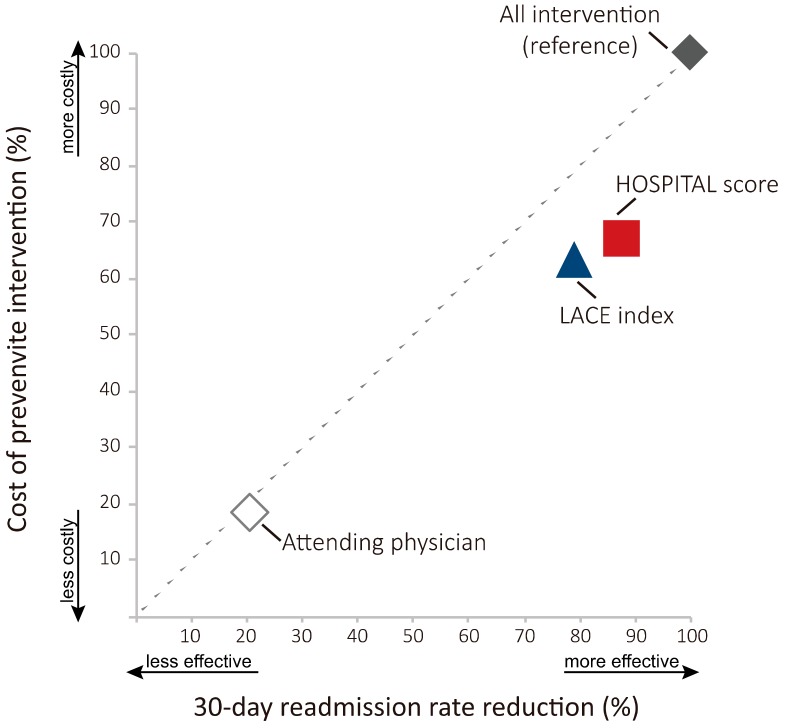
Cost-effectiveness scatter plot of cost of preventive intervention against readmission by 30-day readmission rate reduction of the LACE index, HOSPITAL score, and attending physician models compared to the all intervention model (no prediction, a null model). A simulated result of acute hospitalizations of a home care patient cohort of a medical center affiliated home care unit in Taiwan, 2017 (*n* = 87). The higher 30-day readmission rate reduction implies greater effectiveness in reducing readmission, while the higher cost of preventive interventions implies more expense. The dotted diagonal line refers to equal cost and readmission rate reduction. Different data points denote different prediction models. Dots below the dotted diagonal line (right lower area) are deemed more efficient than dots above the line (left upper area).

Both the LACE index and HOSPITAL score models are more efficient than attending physicians and all intervention (no prediction, null model). When all costs of preventive intervention and 30-day readmission rate reduction are considered together, the all intervention model is naturally the least efficient one since preventive interventions are performed on all hospitalizations; thus, it has the highest cost of interventions and the highest 30-day readmission rate reduction (Figure 4). Taking the all intervention model as a reference, both the LACE index and HOSPITAL score models reduced 30-day readmission rates with a lesser cost of intervention (Figure 4).

## 4. Discussion

The current study assesses the performance of the LACE index and HOSPITAL score models, the two most commonly used prediction models to identify patients at high risk of hospital readmission; moreover, we use real-world data from a cohort of home health care patients to evaluate the real-world effectiveness of these two models as a risk assessment tool prior to execution. Such real-world evidence is important for home care providers and for the evaluation framework that generates real-world evidence which is much more critical for policy makers.

Prediction of readmission is critically important to reduce readmissions for home health care patients, those who are usually old and with certain degrees of disabilities. The results show that the HOSPITAL score was superior to the LACE index in terms of discrimination (the ability to identify hospitalization with a higher probability for readmission). While both the LACE index and HOSPITAL score models effectively reduced readmission rates at 40% compared to attending physicians, the HOSPITAL score model provided slightly better performance than the LACE index. The current study offers clear real-word evidence of the usefulness of the prediction model as a risk assessment tool against readmission for home care patients. Home care providers are encouraged to consider adopting the HOSPITAL score model to optimize their care plans.

Our results further extend the current knowledge not covered in previous studies to home care patients. Both the LACE index and HOSPITAL score demonstrate convincing clinical values in reducing readmission even though the discrimination power of these models seems suboptimal. This finding is similar to other works summarized in Appendix B. We reviewed 23 previous works and found that the discrimination power of either the LACE index or HOSPITAL score only reached ‘poor’ to ‘possibly helpful’ level but was still useful in most of the scenarios (AUC of LACE index ranges 0.55–0.82 in Table A3 vs. AUC of HOSPITAL score ranges 0.60–0.77 in Table A4). Moreover, while LACE index and HOSPITAL scores previously focused on the relative health population, the current results add new information about a novel use of prediction models as risk assessment tools in an old (mean age is 87.4 years old) disabled home care population with high comorbidities (average comorbidities index = 1.7) and high mortalities (mortality rate >20% per year).

The results clearly demonstrate that the readmission prediction model is a useful risk assessment tool for home care patients. Our results reveal a 40% reduction in the readmission rate by proactively targeting preventive intervention toward high-risk hospitalizations of home care patients. Previous studies also showed that a great portion of 30-day readmissions are avoidable through a series of resource-intensive interventions, including post-acute support, post-discharge monitoring [24], telehealth rehabilitation programs [25], and home care case management [26,27]. A readmission prediction model is of course the very first step to stratify patients and optimize resource utilization for home care patients. Moreover, a growing body of evidence suggests an earlier use of readmission prediction models prior to discharge. Readmission prediction made during hospitalization would help the home care team to prepare coaching sessions to care givers, transitional care intervention programs [28,29], and to arrange supporting services including social workers, in-home rehabilitation, and in-home nutrition consultation, before the patients are discharged to their homes and has the potential to prevent more readmissions [30,31].

The current study presents a comprehensive framework to evaluate the real-world effectiveness of prediction models. An evaluation of the performance of predictive models is never an easy task. Traditionally, a dozen performance measures such as sensitivities, specificities, a confusion matrix, ROC curves, AUC (also known as c-statistics), etc. have been proposed; however, none of these measures sufficiently reflect the effectiveness of models, so that we cannot compare models using any of these measures alone [19]. To address this issue, the net reclassification index (NRI) is a novel method trying to provide a single-number summary for assessing how much better a new model is at predicting risk than another model [20]. However, the NRI is not without problems and may be misleading in certain conditions [32]. We thus built a microsimulation model based on real-world data to better estimate the cost and effectiveness in the real-world [21]. Our analytic framework, combining traditional measures, NRI, and a microsimulation model, would be particularly useful in implementing prediction models in clinical practice. For example, prediction model developed in a large research sites could be adopted and applied to other clinical settings by incorporating this framework [33,34].

Both the LACE index and HOSPITAL score prediction models show a satisfactory reduction in readmission rates; however, there is still room for improvement for these models. While readmission is the common endpoint resulting from multiple factors [35,36], some previous studies suggested a limited use of the LACE index and HOSPITAL score models because these two models include only a few variables which lead to inconsistent performance across different settings [7,12,37,38]. Moreover, the current study found only a portion of variables have predictive power for readmission among home care patients (one out four variables in the LACE index, three out of six variables in the HOSPITAL score, respectively). Therefore, some studies suggested introducing more variables or using state-of-the-art methods such as machine learning and deep learning to gain a better predictive power [39,40,41]. However, using the real-world data, we found that both the LACE index and HOSPITAL score models already perform far better than physicians and might be good enough in readmission rate reduction for home care patients. The predictive power of a model is not everything; our results suggest real-world evidence generated from simulation using real-world data would help decision-makers in choosing a prediction model with more clinical usefulness [18,42].

Prediction models should augment home care team’s decision making, not replace them. Ethical considerations should never be overlooked when putting prediction models into practice. Previous studies on anchoring effects suggested that the process of human decision making is easily influenced by an initial piece of information (LACE index or HOSPITAL score in the current study), which is hard to avoid [43]. Moreover, a certain portion (10% to 20%) of patients with high risk for readmission would be falsely stratified as low risk using either the LACE index or HOSPITAL score model. As a result, we suggest that these prediction models should never be used alone. Furthermore, special attention and auditing should be made to patients stratified as low risk.

Future studies will have to look at ways to develop a more accurate and generalized prediction model for home care patients. Many parameters are not fitted well for home care patients and Taiwan’s health system. For example, the length of stay (L) in Taiwan is usually much longer than other health systems, home care patients are often admitted via the emergency department but the outpatient department (acuity of admission (A)), and those patients are rarely admitted to the oncology ward for cancer treatment (discharge from an oncology service (O)). Moreover, the LACE index and the HOSPITAL score take different parameters into consideration, and it is possible that these two models may be appropriate for home care patients that are of different conditions. As a result, a new model combining the LACE and HOSPITAL models with re-calibration according to Taiwan’s health system may increase the predictive power for home care patients. Furthermore, pooling validating results from other sites externally would be important before extrapolating the current results.

There are some limitations in the current study. First, generalizability is the fundamental issue of prediction models. Although the current study clearly demonstrated the feasibility of using the LACE index and HOSPITAL score models as risk assessment tools for patients in a large home care facility in Taiwan, more data from home care facilities of different scales are needed to ensure the result is applicable to every home care facility. However, the current study presents a comprehensive framework so every home care facility can validate the result using their own data and make the result more applicable. Secondly, system level factors for readmission are not considered in the current study. Although both the LACE index and HOSPITAL score models worked well in the current setting, the results may deviate when there is system change in insurance policy, coverage of home care service, accessibility of hospital services, etc. Therefore, these prediction models as risk assessment tools should be validated and calibrated periodically. Thirdly, many parameters of both models are not fitted well for home care patients and Taiwan’s health system. This decreases the predictive power of these prediction models. A revised prediction model based on the LACE index and HOSPITAL score models might have better performance. Fourthly, the current prediction model is a simplification of real-life scenarios and the inability to fully model the complex nature of readmission. As in our results, the strength of the readmission rate reduction is dependent on the choice of model structure. Rigorous model validation and assessment of the performance and outcomes can help assess the robustness of the model findings and address this limitation.

## 5. Conclusions

While both the LACE index and HOSPITAL score models effectively reduce the readmission rate, the HOSPITAL score model shows a slight advantage over the LACE index model as a risk assessment tool for home care patients. Rigorous validation using a simulation model based on local real-world data is warranted before generalization and execution of the current result.

## Figures and Tables

**Table 1:** Demographic information and parameters used in the LACE and HOSPITAL prediction models by outcomes of acute hospitalization of a home care patient cohort of a medical center affiliated home care unit in Taiwan, 2017 (*n* = 87).

Demographics and Parameters	All Acute Hospitalizations (*n* = 87)		Acute Hospitalization Followed by 30-Day Readmission (*n* = 22)		Acute Hospitalization with Successful Discharge (*n* = 65)		-	-
	Mean	(SD ^1^)	Mean	(SD ^1^)	Mean	(SD ^1^)	*p*-Value	Sig. ^2^
**Personal demographics**								
Gender	-	-	-	-	-	-	0.055	-
Female, no. (%)	40	(46.0)	14	(63.6)	26	(40.0)	-	-
Male, no. (%)	47	(54.0)	8	(36.4)	39	(60.0)	-	-
Age	87.4	(12.4)	89.0	(8.1)	86.9	(13.6)	0.482	-
**LACE prediction model**								
Length of stay (L)	5.6	(0.7)	5.6	(0.6)	2.6	(0.6)	0.654	-
Acuity of admission (A)	2.7	(0.9)	2.9	(0.6)	2.6	(0.6)	0.21	-
Comorbidities (C)	1.7	(0.9)	1.6	(0.9)	1.7	(0.9)	0.864	-
Emergency department visits (E)	1.9	(1.3)	2.5	(1.1)	1.7	(1.1)	0.017	*
LACE index	11.8	(2.6)	12.5	(1.8)	11.5	(1.8)	0.053	-
**HOSPITAL prediction model ^3^**								
Hemoglobin level (H)	0.7	(0.5)	0.9	(0.4)	0.6	(0.5)	0.009	**
Sodium level (S)	0.3	(0.4)	0.1	(0.3)	0.3	(0.5)	0.009	**
Procedure during the index admission (P)	0.3	(0.5)	0.3	(0.5)	0.3	(0.5)	0.967	-
Index type of admission (IT)	0.9	(0.3)	0.9	(0.3)	0.8	(0.4)	0.465	-
Number of admissions (A)	1.7	(1.2)	2.3	(1.2)	1.5	(1.2)	0.007	**
Length of stay (L)	1.9	(0.4)	2.0	(0.0)	1.9	(0.4)	0.083	-
HOSPITAL score	5.8	(1.6)	6.6	(1.4)	5.5	(1.6)	0.003	**

^1^ SD: standard deviation; ^2^ Sig.: significance level: * *p* < 0.05; ** *p* < 0.01; ^3^ The study population rarely uses oncology services and thus the parameter ‘discharge from an oncology service (O)’ is not included.

**Table 2:** Area under curve (AUC), sensitivity, and specificity of the LACE index and HOSPITAL score prediction models for 30-day readmission after acute hospitalizations of a home care patient cohort of a medical center affiliated home care unit in Taiwan, 2017 (*n* = 87).

**30-Day Readmission Prediction Models**	**Area under Curve (AUC)**	**(95% C.I.)**	***p*-Value**	**Sig. ^1^**	**-**
LACE index	0.598	(0.474–0.722)	0.170	-	-
HOSPITAL score	0.691	(0.573–0.808)	0.008	**	-
**30-Day Readmission Prediction Models**	**Accuracy (%)**	**Sensitivity (%)**	**(95% CI)**	**Specificity (%)**	**(95% CI)**
LACE index	54.5	81.8	(59.7–94.8)	44.6	(32.3–57.5)
HOSPITAL score	54.0	90.9	(70.8–98.9)	41.5	(29.4–54.4)

^1^ Sig.: significance level: ** *p* < 0.01.

**Table 3:** Thirty-day readmission rate reduction and cost of preventive intervention by the LACE index and HOSPITAL score prediction models and all intervention model compared to the attending physician model. Simulated results of acute hospitalizations of a home care patient cohort of a medical center affiliated home care unit in Taiwan, 2017 (*n* = 87).

30-Day Readmission Prediction Models ^1^	30-Day Readmission Rate Reduction (%)	(IQR ^3^)	Cost of Preventive Intervention (%) ^4^	(IQR ^2^)
LACE index	39.2	(39.1–39.4)	66.8	(61.0–72.6)
HOSPITAL score	43.4	(43.3–43.5)	72.0	(63.1–79.4)
Attending physician	10.1	(9.8–10.3)	18.6	(17.2–19.9)
All intervention	50.0	(50.0–50.0)	100 (reference)	-

^1^ Preventive intervention was performed depending on the likelihood of 30-day readmission made by each prediction model. LACE index: LACE index ≥11; HOSPITAL score: HOSPITAL score ≥5; attending physician: simulated prediction made by attending physicians [23]; all intervention: preventive efforts are performed for all acute hospitalizations without any prediction. ^2^ IQR: interquartile range. ^3^ The current model only includes the direct costs of preventive intervention against readmission for acute hospitalization for those predicted as at high risk for readmission, thus the cost of a physician (e.g., extra payment) and maintenance of a computer system are not counted. Cost of the all intervention model (i.e., no prediction model) is used as a reference.

## Appendix A

**Table A1:** Summary of parameters used in the microsimulation model to evaluate the 30-day readmission rate reduction and cost of preventive intervention by the LACE index, HOSPITAL score, and attending physician prediction models.

30-Day Readmission Prediction Models	Sensitivity (%)	(95% CI)	Specificity	(95% CI)
LACE index	81.8	(59.7–94.8)	44.6	(32.3–57.5)
HOSPITAL score	90.9	(70.8–98.9)	41.5	(29.4–54.4)
Attending physician ^1^	23	(13–37)	84	(75–90)

^1^ Parameters used for the attending physician model were extracted from [23].

**Table A2:** Summary of variables and key parameters used in the microsimulation model.

Variables	Type, Value	Lower Limit	Mode	Upper Limit
Readmission rate	Constant = 0.25	-	-	-
Successful rate of preventive intervention	Constant = 0.5	-	-	-
LACE index—Sensitivity	Triangular distribution	0.597	0.818	0.948
HOSPITAL score—Sensitivity	Triangular distribution	0.708	0.909	0.989
Attending physician—Sensitivity	Triangular distribution	0.13	0.23	0.37
LACE index—Specificity	Triangular distribution	0.323	0.446	0.575
HOSPITAL score—Specificity	Triangular distribution	0.294	0.415	0.544
Attending physician—Specificity	Triangular distribution	0.75	0.84	0.90

## Appendix B

**Table A3:** Summary of previous studies which used the LACE index predictive model for readmission of different populations. Ordered according to year of publication.

No.	Studies Used LACE Index	Population (*n*)	Area under Curve (AUC)	(95% CI) ^1^	Discrimination Power ^2^
1.	Cotter et al., (2012) [12]	UK population, *n* = 507	0.57	NA	Poor
2.	Van Walraven et al., (2012) [44] ^3^	Medical records, *n* = 500,000	0.771	(0.767–0.775)	Clearly useful
3.	Wang et al., (2014) [45]	Heart failure patients, *n* = 253	0.664	(0.575–0.752)	Possibly helpful
4.	Low et al., (2015) [46]	Singapore population, *n* = 5862	0.628	(0.602–0.653)	Possibly helpful
5.	Yazdan-Ashoori et al., (2016) [47]	Heart failure patients, *n* = 378	0.58	(0.57–0.61)	Poor
6.	Damery et al., (2017) [48]	Inpatient health records, *n* = 7107	0.773	(0.768–0.779)	Clearly useful
7.	Low et al., (2017) [49]	Singapore, elder inpatients, *n* = 17,006	0.595	(0.581–0.608)	Poor
8.	Robinson et al., (2017) [13]	Inpatient health records, *n* = 432	0.58	(0.48–0.68)	Poor
9.	Baig et al., (2018) [50]	New Zealand population, *n* = 213,440	0.752	NA	Clearly useful
10.	Hakim et al., (2018) [51]	COPD ^4^ patients, *n* = 2662	0.63	(0.62–0.65)	Possibly helpful
11.	Miller et al., (2018) [37]	Inpatients, *n* = 359	0.620	(0.521–0.718)	Possibly helpful
12.	Saluk et al., (2018) [52]	Radical cystectomy patients, *n* = 3470	0.581	(0.556-0.606)	Poor
13.	Caplan et al., (2019) [53] ^3^	Brain tumor population, *n* = 352	0.58	NA	Poor
14.	Caplan et al., (2019) [54] ^3^	Craniotomy patients, *n* = 238	0.69	NA	Possibly helpful
15.	Ibrahim et al., (2019) [55]	Heart failure patients, *n* = 730	0.551	(0.503–0.598)	Poor
16.	Robinson et al., (2019) [56]	Inpatients, *n* = 1916	0.598	(0.58–0.64)	Poor
17.	Shaffer et al., (2019) [57]	Surgical patients, *n* = 192,670	0.82	NA	Possibly helpful

^1^ CI: confidence interval, NA: not available. ^2^ Discrimination power: AUC < 0.60, poor discrimination; AUC = 0.60 to 0.75, possibly helpful discrimination; AUC > 0.75, clearly useful discrimination [19]. ^3^ A modified version of LACE index, LACE+ index, is used. ^4^ COPD: Chronic Obstructive Pulmonary Disease.

**Table A4:** Summary of previous studies used HOSPITAL score predictive model for readmission for different populations. Ordered according to year of publication.

No.	Studies Used HOSPITAL Score	Population (*n*)	Area under Curve (AUC)	(95% CI) ^1^	Discrimination Power ^2^
1.	Aubert et al., (2016) [10]	Switzerland, inpatients, *n* = 346	0.70	(0.62–0.79)	Possibly helpful
2.	Donze et al., (2016) [58]	Inpatients, *n* = 117,065	0.72	(0.72–0.72)	Possibly helpful
3.	Kim et al., (2016) [59]	Inpatients, *n* = 4208	0.65	NA	Possibly helpful
4.	Robinson (2016) [60]	Inpatients, *n* = 998	0.77	(0.73–0.81)	Clearly useful
5.	Aubert et al., (2017) [61]	Inpatients, *n* = 117,065	0.69	(0.68–0.69)	Possibly helpful
6.	Burke et al., (2017) [8]	Inpatients, *n* = 9181	0.68	(0.67–0.71)	Possibly helpful
7.	Robinson et al., (2017) [13]	Inpatient health records, *n* = 432	0.75	(0.67–0.83)	Possibly helpful
8.	Ibrahim et al., (2019) [55]	Heart failure inpatients, *n* = 730	0.595	(0.549–0.641)	Poor
9.	Robinson et al., (2019) [56]	Inpatients, *n* = 1916	0.675	(0.65–0.70)	Possibly helpful

^1^ CI: confidence interval, NA: not available. ^2^ Discrimination power: AUC < 0.60, poor discrimination; AUC = 0.60 to 0.75, possibly helpful discrimination; AUC > 0.75, clearly useful discrimination [19].
